# Prof. Mišo Mihajlo Virag, MD, PhD, FRCS - a guiding light faded away (9.07.1946–16.08.2018)

**DOI:** 10.1186/s40902-018-0166-0

**Published:** 2018-09-12

It is with great sorrow that we commemorate the loss of our great mentor and friend, Professor Mišo Mihajlo Virag, MD, PhD, FRCS (Eng). For those of us who knew him, this is and always will be an irreplaceable loss!

**Fig. 1:**
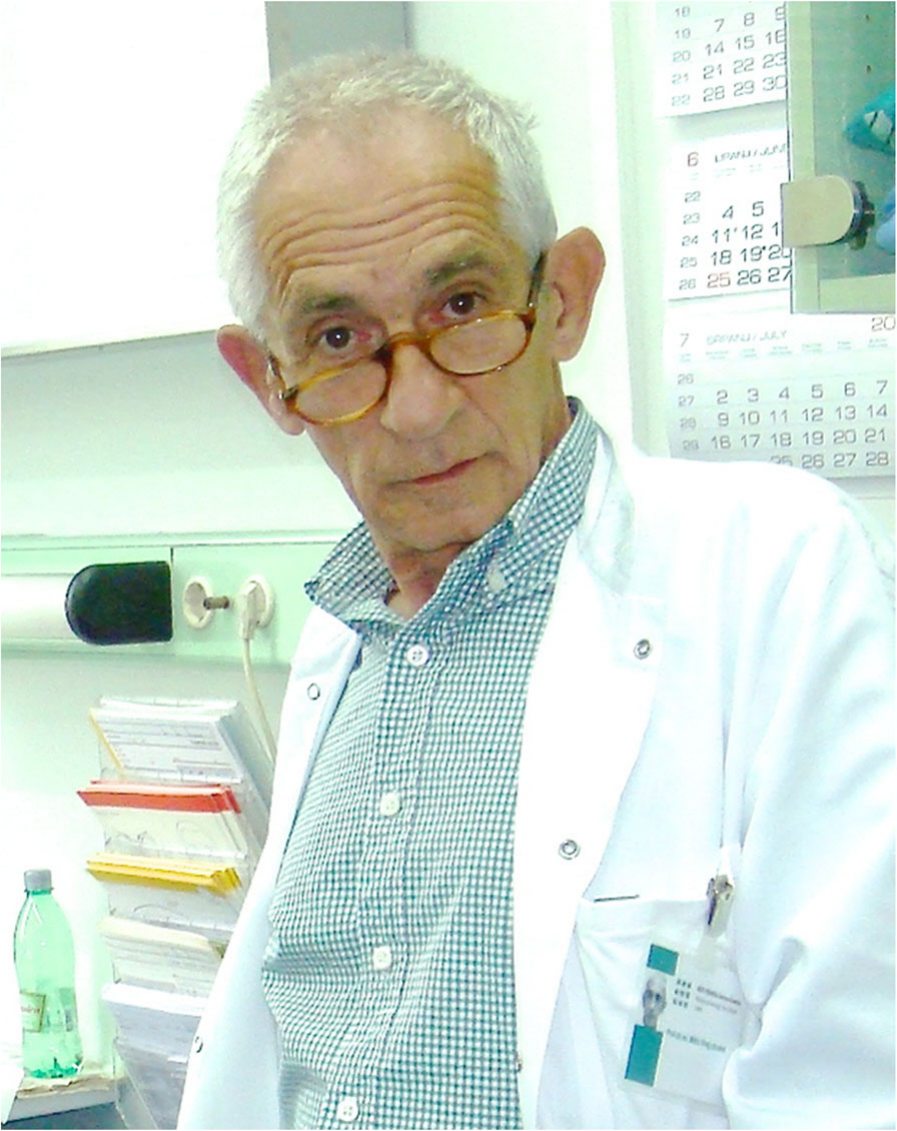
Prof. Mišo Mihajlo Virag

Born on the 9th of July 1946, in Zagreb, in a distinguished family of physicians, Prof. Mišo Virag embraced medicine with passion, graduating from the Medical School of Zagreb University in 1971. He was initially certified in Otolaryngology and Cervicofacial Surgery and, immediately after that, in Maxillofacial Surgery, becoming a revered Head and Neck Surgeon.

It came as no surprise that he was soon to be accepted for head and neck surgery training in Sloan Kettering Memorial Cancer Centre New York and MD Anderson Cancer Center, Houston, Texas, as well as other important oncological centres in Europe (Canniesburs Hospital, Glasgow, Royal Marsden Hospital, London, Hospital Lariboisiere, Paris). Therefore, we can truthfully say that Prof. Mišo Virag received an outstanding education in oncology. At about the same time, he also started a long lasting friendship with world-renowned Prof. Jatin Shah.

Professor Virag was the Head of the Department of Maxillofacial Surgery in University Hospital Dubrava, Zagreb, Croatia and the Chair for Maxillofacial Surgery in the Medical School of Zagreb University. He had been appointed as Visiting Professor in many European or international universities in countries like Zurich, Switzerland, Budapest, Hungary, Shreveport, Louisiana, and the USA. He was a distinguished member of several international societies, but above all, he was the President of the European Society for Cranio-Maxillofacial Surgery (2010–2012).

He was also very active as a worldwide invited lecturer, not only under the auspices of EACMFS or very renowned congresses but for local courses as well. He was not a man to enjoy or even search the spotlight but he was deeply convinced of his mission of sharing his knowledge with young surgeons and spreading knowledge in the interest of the speciality.

In these positions, he was mentoring many generations of surgeons, including myself. Since 2004, when he started his lectures within the framework of EACMFS Romanian Educational Rolling Program, I was fortunate enough to benefit from his professional wisdom and skills.

He was also deeply involved in clinical and fundamental research, mainly in the area of oral cancer. It is worth mentioning that he published a great number of important articles and conference presentations. It is Professor Jatin Shah who mentioned Prof. Mišo Virag as “one of the hundreds of men and women, who made important contributions in head and neck cancer in the past century” (Jatin P. Shah: *A Century of Progress in Head and Neck Cancer.* Jaypee Brothers Medical Publishers, Philadelphia 2014).

Most of all, Professor Virag was a relentless surgeon, travelling all over the world to save lives. As he was always saying, he was not difficult to convince to fly to whatever point on the world map to be involved in the treatment of a difficult case. And he did this up to the very end. For this reason, we, the Eastern European colleagues, are especially in debt to him.

As a very modest personality, he never bragged about his professional accomplishments and about the honours and worldwide recognition he received. Nor did he let anyone feel his superiority. It is not very often that one gets the privilege of meeting a person who is so powerful but also humble at the same time, as Mišo Mihajlo Virag.

He was as passionate about sailing as he was for oncology. It is the reason why he won many Croatian regattas. For him, saving lives was not an attitude belonging only to the operation theatre. He extended his life-saving skills at the sea as well, for which he received the “Blue Ribbon” Award with the crew of the Munjek sailing boat. In his effort to harmonise his profession with his hobby, Professor Virag was the soul of “Maxillofacial International Sailing Tour” on the Adriatic Sea, which had his 22nd edition in 2018. Numerous European maxillofacial surgeons took part in this sailing trip, holding irreplaceable memories about the time spent with Mišo Mihajlo Virag.

Mišo Mihajlo Virag was the kind of man who liked to “burn the candle at both ends”, living to help his patients and to pursue his passions. Sadly enough for all of us, on a quiet August afternoon, Prof. Mišo Virag decided to go sailing forever, exploring the infinite sea. The same day, we, all the people who knew him, lost an ocean.

Horatiu Rotaru, DMD, MD, PhD

Assistant Editor

